# Iterative hybrid model based optimization of rAAV production

**DOI:** 10.1002/btpr.70006

**Published:** 2025-03-24

**Authors:** Claudio Müller, Gerald Siegwart, Susanne Heider, Michael Sokolov, Angela Botros, Alexandra Umprecht, Moritz von Stosch, Mariano Nicolas Cruz Bournazou

**Affiliations:** ^1^ DataHow Zurich Switzerland; ^2^ Pharmaceutical Sciences, R&D Baxalta Innovations GmbH, a Takeda company Vienna Austria; ^3^ Technische Universität Berlin Institute of Biotechnology, Chair of Bioprocess Engineering Berlin Germany

**Keywords:** Design of Experiments, human embryonic kidney suspension cell, hybrid modeling, Parallel Mini‐bioreactors, rAAV production

## Abstract

Changes in serotype or genetic payload of recombinant adeno associated virus (rAAVs) gene therapies require adapting the transfection conditions of the upstream HEK293 cultivations. This study adopts an iterative model‐based experiment design approach, where increasing data availability is leveraged to evolve models of different complexity. Initial models based on data from shaker flask runs guided the design of the first round at Ambr250 scale. With Ambr250 data becoming available, hybrid models capturing process state evolutions and historical models incorporating these evolutions to predict rAAV titer, were developed. These models were then combined into a full model approach, which was utilized within a Bayesian Optimization framework for the design of a second round of Ambr250 scale runs. The iterative approach was tested across different projects applying transfer learning to enhance the predictive power and improve the subsequent optimization. The approach was benchmarked against a statistical Design of Experiment method. The results show that the model‐based experiment design consistently (and across projects) produces higher rAAV titer values than the benchmark approach (Project C: 4.4% or 7.0% increases in titer values relative to the response surface modeling approach for ELISA and ddPCR, respectively; Project D: 32.4% or 10.9% increases in titer values relative to the standard DoE‐screening pick for ELISA and ddPCR, respectively), effectively optimizing the transfection mixture composition. The combination of propagation and historical models, augmented by transfer learning and an ever‐increasing amount of data, enhanced the process design workflow, contributing to improved rAAV production through efficient transfection strategies.

## 1 INTRODUCTION

The field of gene therapy has come a long way and has experienced a renaissance in recent years.^1^ Recombinant adeno associated virus (rAAV) based gene therapy has become the leading platform for in vivo gene transfer.^2^ Engineering of the rAAV vector can further increase its potential^3^ and might make it amenable to treat a larger population.^1^

Production of rAAV is typically accomplished with human embryonic kidney suspension cell (HEK293) cultivations (though originally adherent, the cell line was modified to allow for cultivation in suspension, bring along several advantages, see e.g.,).^4^ The upstream process can be divided into two phases: cell expansion phase and production phase. Cell expansion occurs in bioreactors of increasing volume to finally reach production scale followed by transient transfection of the cells using plasmids and transfection reagent. Transfection initiates the transition to the production phase: After the plasmid‐DNA is taken up by the cells, rAAV is produced. The genetic payload (therapeutic gene) is enclosed within the produced rAAV capsids. Ideally, all capsids contain the full‐length payload, but nonetheless a large proportion of capsids produced are either empty or only partially filled that is, with a truncated DNA payload.

Both the rAAV vector and payload are modified to target different diseases and tissues inside the body. Therefore, optimization of the upstream production conditions become necessary (i) to ensure highest possible overall capsid quantities while (ii) still considering maximizing the proportion of full‐length filled capsids and minimizing the number of empty capsids. This optimization step requires the execution of several runs usually conducted in a design of experiment (DoE) setup and therefore typically carried out in small scale to reduce costs and timelines. Regardless, (partially) empty capsids are being produced, process understanding remains limited and with the advent of new therapeutic genes, optimizations must be performed anew.

Mathematical modeling of the production process is expected to alleviate these challenges. For HEK293 cultivations, several metabolic and process models have been developed.^5^, ^6^, ^7^ A recent study proposes a model of the transfection process,^8^ starting from exogenous DNA delivery to the reaction cascade that forms viral proteins and DNA (full capsids) as well as the Rep protein (a regulator of the packaging plasmid gene expression and a catalyst for viral DNA packaging). A review paper on process modeling of rAAV production in HEK293 cells^9^ suggests that hybrid modeling approaches might prove successful in describing the process behavior even for different capsids and serotypes.

In what follows, we describe the experimental set‐up, generation of process run data for different projects, the process modeling approaches that were utilized to describe process behavior for shaker‐flask and small scale stirred reactors as well as the optimization procedure. The objective was maximizing rAAV titer, particularly genomic titer (ddPCR, full capsids) and ELISA (all capsids). Subsequently, we present the modeling results, the outcome of the iterative process optimization within different projects as well as the result of transferring the process modeling across projects (different payloads). The results of different modeling approaches are compared, namely using traditional approaches, such as Response Surface Modeling compared to the novel Full Model methodology that includes process dynamics predictions with hybrid models. In the end, we conclude that the proposed modeling and optimization approach can be used to decrease the experimental effort and increase the understanding of the process behavior.

## 2 METHODOLOGY

### 2.1 Process, analytics and data

#### 2.1.1 Process description

##### Seed train

A strain‐stock of proprietary HEK293 cell line adapted to suspension growth was cultivated in chemically defined media (Thermo Fisher, NY, USA) at 37 °C in HERA Cell 150 incubator (Thermo Fisher Scientific, NY) in humidified atmosphere. Cell expansion was performed in disposable spinner flasks of increasing volume (Corning, Germany) by splitting the cells each 2 to 3 days.

##### Ambr250 runs

After cell expansion, cells were transferred to an Ambr® 250 high throughput (HT) fully automated microbioreactor system (Sartorius, Germany) under controlled conditions (pH, CO_2_, O_2_). Vessels were inoculated to reach a final density of 0.5 × 10^6 cells/mL, as measured using a NucleoCounter NC‐200 (Chemometec, Denmark). Cells were allowed to grow to approximately 7.5 × 10^6 cells/mL. On the day of transfection, cultures were adjusted to 4 × 10^6 cells/mL before proceeding with the transfection procedure. Transient rAAV production was initiated with triple transfection (focusing exclusively on the AAV9 serotype) that is, three plasmids combined in a single transfection procedure. The Helper‐plasmid (HLP) is containing the necessary Adenovirus 5 Helper genes, AAV9 Rep‐Cap plasmid (RC) accounts for rAAV serotype specificity and required packaging genes while a Transgene‐plasmid (TG) delivers the gene of interest including the required promoter, flanked by ITR sequences. Transfection procedure was carried out with Polyethyleneimine (PEI) (Polysciences, PA) as transfection reagent. The transfection mixture was prepared by sequentially adding plasmids to the media, mixing for 30 s, then adding PEI. After brief mixing, complexation time was 25 min without agitation. After transfection, cells were cultivated using the Ambr® 250 HT system with daily measurements of metabolites and rAAV‐titers. After cultivation, samples were subjected to cell lysis via a single freeze/thaw cycle. 15 mL samples were frozen at −80 °C overnight, then thawed, mixed, and centrifuged. The supernatant was aliquoted for subsequent analytics.

##### Shaker Flask Runs

Some experimental runs were performed using the HEK293 suspension cells in shaker flasks (Corning, New York). The inoculation, cell density adjustment, transfection procedure, and lysis method were carried out under the same conditions as described for the Ambr system. After transfection, shaker flasks were placed in a New Brunswick S41i incubator (Eppendorf, Hamburg, Germany). Production was monitored with end‐point measurement of rAAV titers.

#### 2.1.2 Analytics

Substrates and metabolites including glucose, lactate, glutamine, and glutamate were determined with a Cedex Bio HT Analyzer (Roche Diagnostics, Germany). Cell viability and cell density were determined using a NucleoCounter® NC‐200™. Analytics for viral titers were performed using in‐house methods (ELISA and ddPCR).

ELISA: The sandwich‐ELISA‐method targeting the capsid of the respective rAAV‐serotype is used routinely in‐house to quantify capsid production and purification. Each sample is analyzed four times in a 1:2 dilution series whereof the titer is calculated. Blank and control samples are included on each plate to ensure consistency as well as uncontaminated reagents. For accuracy, spiking runs were done that show a recovery of >90% to <105%. Precision (repeatability) testing shows a coefficient of variation (CV) below 2.5% (inter‐ and intra assay; data not shown). For campaign C1, samples were manually prepared using the washing device Biotek 405LS, whereas for the remaining campaigns the automated Hamilton Vantage liquid handler was used.

The used droplet digital PCR (ddPCR) method is amplifying two different sequences within the capsid‐DNA (duplex‐ddPCR). One target sequence is located in the ITR region that is flanking the gene of interest while the second target is a proprietary sequence‐Tag outside the gene of interest. Residual DNA (plasmids etc.) are digested in sample preparation phase, to not affect titer measurements. Accuracy was tested between 95% and 117% for both sequences in the relevant matrix. Regarding precision (repeatability), a CV <6% was found for the relevant matrix for both targets (data not shown). For all campaigns, the samples were prepared using Hamilton Vantage liquid handler and analyzed using QX ONE (Bio Rad, Hercules, California).

#### 2.1.3 Process runs and data

Two different projects, project C and project D, were part of this study. For each of them, two campaigns were performed in the Ambr scale. A campaign consisted of up to 16 runs being performed concurrently in the Ambr250 HT system. The campaign label C1 refers to the first set of runs in the Ambr scale of project C, C2 to the second iteration, and identically for project D.

##### Initial shaker flask runs

The initial data comprised 25 shaker flask runs for project C and D, each. Among these runs, factorial combinations of three primary design parameters HLP, RC and TG plasmid molarities were executed in duplicates, and the center point was run in triplicates. Another design parameter, transfection reagent to plasmid ratio, was also varied for a separate condition outside of the factorial combinations. Additionally, the analytics data for viral titer (ELISA and ddPCR) were available. For the shaker flask runs, data of the cell density, viability as well as metabolite concentrations were not available.

##### Ambr250 runs

All subsequent campaigns were successively performed in the Ambr scale. The experimental conditions were designed with various approaches that are elaborated in the results section below. To gain information on variability within a campaign and across campaigns, as well as to evaluate the relative performance of the DoE approaches relative to a fixed benchmark, the center point was run as a duplicate in all campaigns. At the Ambr scale, measurements of cells (viable cell density (VCD), viability (Via) and cell diameter (Diam)), key metabolites (glucose (Glc), glutamine (Gln), glutamate (Glu), ammonia (Amm) and lactate (Lac)) and osmolality were performed every day, resulting in 5 total measurements for the growth phase and 4 measurements for the production phase. For each run, analytics for viral titers were at the least performed for the end‐point of the process. For all Ambr runs, pH, temperature, stirring rate and DO were controlled around the same set point, therefore introducing no additional variation to the data.

##### Data partitioning for process modeling

For campaigns C2 and D2, a training and test set were specified, where the models were trained on the training data and the generalization properties were assessed on the test data. The representativeness of the selected test data was assessed prior to the modeling exercise by performing a Principal Component Analysis (an overview and detailed description of PCA can be found here, [10, 11] respectively) on data of VCD, glucose, glutamine, glutamate, ammonia and lactate and visually assessing the similarity of the evolutions by plotting the scores. Further details can be found here [12].

### 2.2 Screening DoE

For all campaigns and in line with the design used for the initial runs, a DoE using a factorial screening design was made and used to compare to the mbDoE approach as well as to manually define the benchmark optimal conditions. The screening DoE used the design parameters (HLP‐, RC‐, and TG‐plasmids, and the transfection reagent ratio TRR) as inputs to estimate the changes in ELISA and ddPCR values. These four design parameters are henceforth called Z0, Z1, Z2, and Z3, and their order is changed to preserve anonymity. All of them have been shown in literature to play a critical role in rAAV production and are therefore considered as suitable parameters to optimize and compare the DoE approaches presented.^13^, ^14^, ^15^, ^16^ The screening DoE was built in MODDE, where the benchmark conditions were also selected manually to find a setpoint that shows high yield together with low plasmid usage.

### 2.3 Response surface modeling

For campaign C2, four conditions were designed using Response Surface Modeling. These types of models are used in traditional approaches and commercial software, therefore representing a meaningful benchmark compared to the Hybrid Model based approaches. In contrast to the latter, Response Surface Modeling does not factor in the process evolution as features, rather only the design parameters *Z*. More details on the model structure can be found elsewhere.^17^

### 2.4 Shaker flask models

For the shaker flask scale, data of the design parameters, *Z*, were known as well as ELISA and ddPCR measurements at the end of the cultivation. Given that data for the evolution of the cultures are not available the objective is to link changes in the design parameters to changes in the process response (ELISA and ddPCR) by using relatively simple models, that is, decision trees (details about this method can be found here [18]) and an Elastic Net model (details about the method can be found here [19]), which allows for feature selection and to manage multicollinearity among features. The Decision Trees were run with maximum depth of 4 and minimum number of four samples in the split. Leave‐one‐out‐cross‐validation (LOOCV) was used for the Elastic Net model. Cross‐validation is a method used for model evaluation. It is an out‐of‐sample testing and is used to estimate the model's ability to generalize to new, never seen data.^20^ LOOCV is a special form of cross‐validation. It is used here, as the dataset is small. For all cross‐validations, replicate experiments are treated as the same entity, and never split across train and validation set. The model hyperparameters were found using a grid search. Overfitting was avoided, using cross‐validation. For all shaker flask models, the Python scikit‐learn version 1.2.2. was used.

### 2.5 Optimization of Ambr runs with shaker flask models

Due to the simplicity of the linear models, two runs in campaign C1 were designed manually. The objective of the optimization was to maximize ddPCR titer. In addition, for campaign C1, a Bayesian optimization approach was adopted that utilizes a Gaussian process model (details about this method can be found here [21, 22]). As acquisition functions, the UCB (upper confidence bound) and the EI (expected improvement) utility function were used to generate one suggestion each for the next experiment designs. In the case of campaign D1, the runs were designed using the decision trees, as described in the results section.

### 2.6 Propagation model

The propagation model describes the evolution of the process over time, that is, the evolution of state variables *X* (VCD, glucose, glutamine, glutamate, ammonia and lactate) as a function of the design parameters *Z*. The dynamic material balance derived for an ideally mixed reactor provide the backbone to the model:

(1) $$\frac{\mathrm{dx}}{\mathrm{dt}}=r-D∙\left(x-x_{f}\right)$$

where *x* is the vector of state variables, *t* is the time, *r* is the vector of reaction rates, *D* is the dilution rate and *x*_*F*_ is a vector of feed concentrations. The reaction rates are each modeled by a Gaussian process model (an overview and detailed description of Gaussian process models can be found here [23, 24] and,^25^, ^26^ respectively), which is trained individually for each reaction term. An Euler forward numerical integration is used, wherefore the prediction of the model at each time step only depends on the last observation state, predefined process setpoints and previous online/offline measurements, that is, *x*_*ti*+1_ = x_*ti*_ + *dx/dt*_*ti*_ ∆*t* and *r*_*j*,*ti*_ = *r*(*x*_*ti*_,*Z*) for each reaction term *j*. A bootstrap sampling approach is used to capture the compounded uncertainties, using an ensemble of 10 models. A more detailed description of the modeling and training methodology can be found here [27, 28, 29].

### 2.7 Historical model

The historical model considers the process evolution (history) besides the control and design parameters to predict the final process attributes *Y*, such as ELISA and ddPCR. The matrices of *X* for this purpose are batch‐wise unfolded (indicated by the indices *flat*) and one model for each attribute *j* was developed, that is:

(2) $$Y_{j}=f\left(X_{\text{flat}},Z\right)$$

with *f*(·) modeled by a Partial Least Squares (PLS) model (an overview and detailed description of PLS models can be found here [30, 31] and,^32^, ^33^ respectively), accounting for the high collinearity of the inputs. An obvious limitation of this approach is that all runs must have the same length, which however in this study is naturally the case. Further, the entire time evolution of the state variables *X* is required, wherefore the propagation model is used in combination with the historical model (referred to in the following as full model) for process optimizations.

A more detailed description of the method can be found here [29, 34].

### 2.8 Optimization with full model

In case of campaigns C2 and D2, the primary objective was the maximization of ddPCR and ELISA titer, that is, max_*Z*0,*…*,3_{*Y*} by manipulation of *Z*_0_ to *Z*_3_. As several runs could be accomplished in parallel, additional designs were proposed by (1) using an expected improvement acquisition inspired objective function,

(3) $$\max_{Z}\left\{\left(Y\left(Z\right)-Y_{\mathrm{opt},1}-\lambda \right)∙\psi \left(u\right)+\sigma_{Y}\left(Z\right)∙\phi \left(u\right)\right\}$$

and increasing the *λ* values (with *Y*_*opt*,1_ the optimal value obtained for the first optimization, *σ*_*Y*_ (*Z*) the standard deviation of the ensemble predictions, *ψ*(*u*) and *ϕ*(*u*) the Cumulative Distribution and Probability Density Functions, respectively, where *u =* (*Y*(*Z*)−*Y*_*opt*,1_)/ *σ*_*Y*_ (*Z*)); and (2) stepwise increasing the time‐averaged risk of misprediction *R* until different process conditions were obtained. The time‐average risk was obtained by (1) training a one‐class support vector classifier on the training data or each campaign; (2) evaluating the trained classifier on each time‐point of the suggested process conditions to obtain the degree of “membership”, *m*(*X*(t),*Z*); and (3) computing the time averaged risk, $R=\frac{1}{t_{r}}\int_{t_{0}}^{t_{\mathrm{end}}}\left(1-m\left(t\right)\right)\cdot \mathrm{dt}$.

Further details can be found here [18, 27, 35]. The optimization problem was solved using a Bayesian optimization algorithm for campaigns C2 and D2. The full model was used during optimizations in C2 and D2. Since the initial conditions, that is the initial state variable values *X*(*t*_0_) cannot be chosen freely, initial conditions of reference runs were used as a starting base of the propagation model. Two reference runs were used, namely the highest and second highest titer yielding runs.

### 2.9 Error metrics

For a quantitative evaluation of the model performance the Mean Squared Error (MSE) is used,

$$\mathrm{MSE}=\frac{1}{N}\sum_{i=1}^{N}{\left(Y_{i}-{\hat{Y}}_{i}\right)}^{2},$$

with *Y*_*i*_ the measurements of variable *Y*, ${\hat{Y}}_{i}$ the model estimate and *N* the total number of measurements, as well as the relative Root Mean Squared Error (rRMSE), that is,

(4) $$\text{rRMSE}=\frac{1}{\sigma_{Y}}\sqrt{\mathrm{MSE}},$$

with *σ*_*Y*_ the standard deviation of the measured variable *Y*.

## 3 RESULTS

**FIGURE 1:**
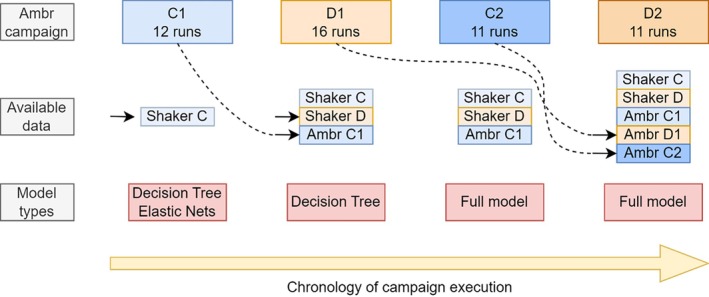
Chronologic order of all four campaigns conducted at the Ambr scale. For each campaign, the available data at the point of creating the design is highlighted. The number of runs that were successfully performed are specified in the top row. The model types used in the design of a campaign are shown at the bottom in the red boxes.

An overview of the relative relation, information and data available for each campaign is shown schematically in Figure 1. As can be seen, the ambition was to make use of available data/knowledge in order to improve process understanding and subsequent designs/optimizations. The black arrows indicate the arrival of new data of previous campaigns. Due to time constraints in analytics, data of the previous campaign was not always available for the next. For instance, the analytics of D1 were not finalized when designing campaign C2. The full model approach was only used from campaign C2 onwards, as it required the full process, dynamics of at least one Ambr campaign of the same project for model training. This is why for campaigns C1 and D1 decision trees and elastic nets were used, as only shaker flask data was available, which is comprised only of the parameters *Z*, as well as the targets ddPCR and ELISA. For each Ambr campaign, the number of runs is shown below the campaign name. Note that for campaign C1 16 runs were planned in total, but 4 bioreactors encountered technical difficulties, which required their removal from the dataset. Campaign D2 was designed for 12 runs, however one run was removed due to technical problems. In what follows, for each campaign the modeling and model‐based design/optimization steps are being described.

### 3.1 Campaign C1: From linear models and decision trees to optimal conditions

#### 3.1.1 Linear models and decision trees

**FIGURE 2:**
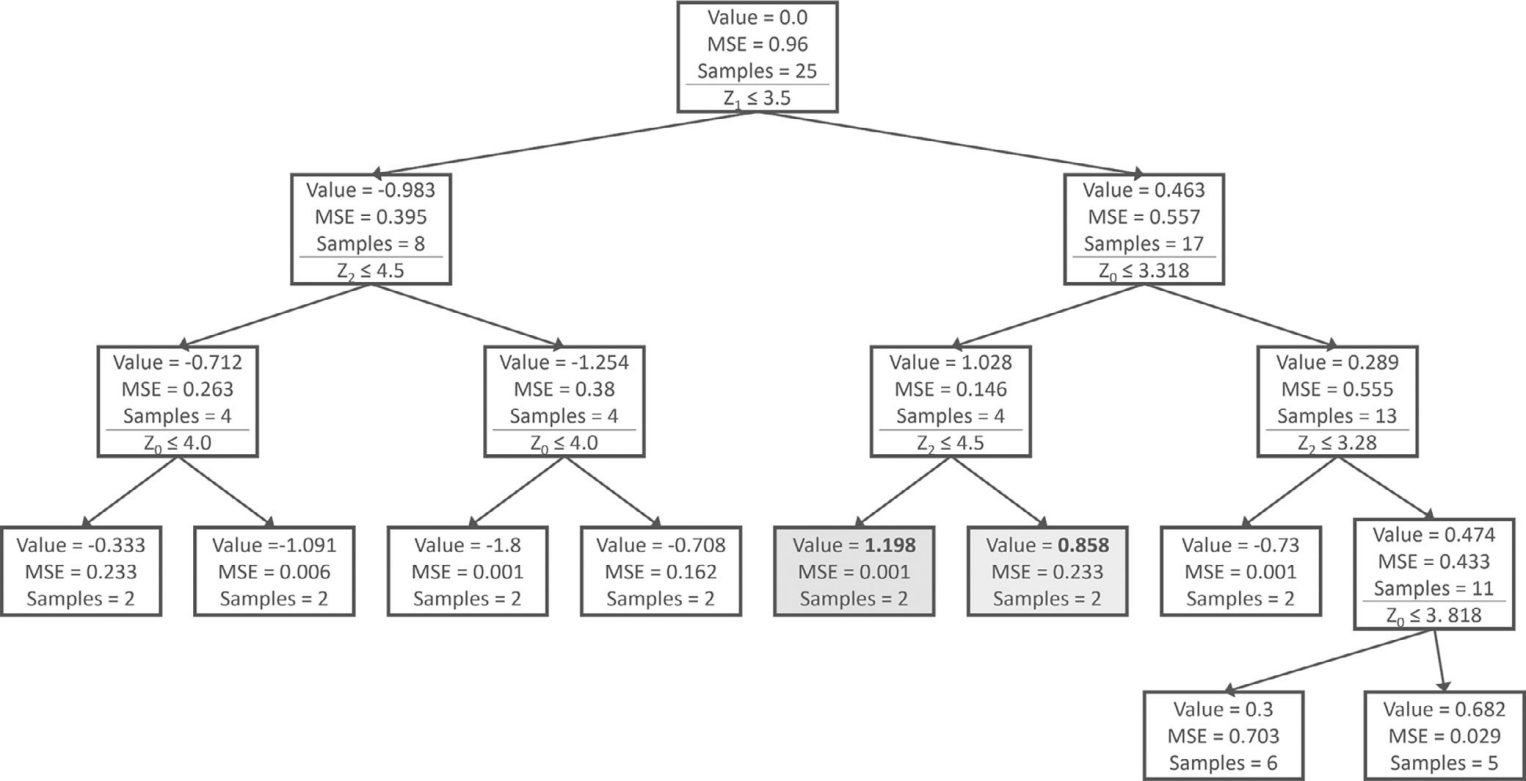
Decision tree model on shaker flask runs of project C targeting ddPCR.

Decision trees were generated to gain insights into the importance of the process design parameters (Z) and their ranges. Figure 2 shows the obtained tree. Every box represents a node in the decision tree. The mean ddPCR value, the MSE, and the number of runs (samples) in each node are reported. The ddPCR value is Z‐score normalized. Below these numbers, the decision split is shown. The left branch evaluates to true, the right branch to false. The nodes with the highest and second highest achieved values are highlighted. *Z1* is the first decision variable. The highest values for ddPCR are obtained when *Z1* is greater than 3.5. Among the remaining runs, *Z0* lower than 3.318 results in high ddPCR. For the best runs, additionally, *Z2* is lower than 4.5. Even though clear conclusions are not possible at this point, the decision tree results hint at the following to be generally beneficial: high *Z1* and low *Z0*, whereas *Z2* has a less clear impact. This is in line with expectations from the shaker flask data, as the corner points of the project C shaker flask data that were simultaneously at the upper and lower bound of *Z1* and *Z0*, respectively, had high ELISA and ddPCR values.

**FIGURE 3:**
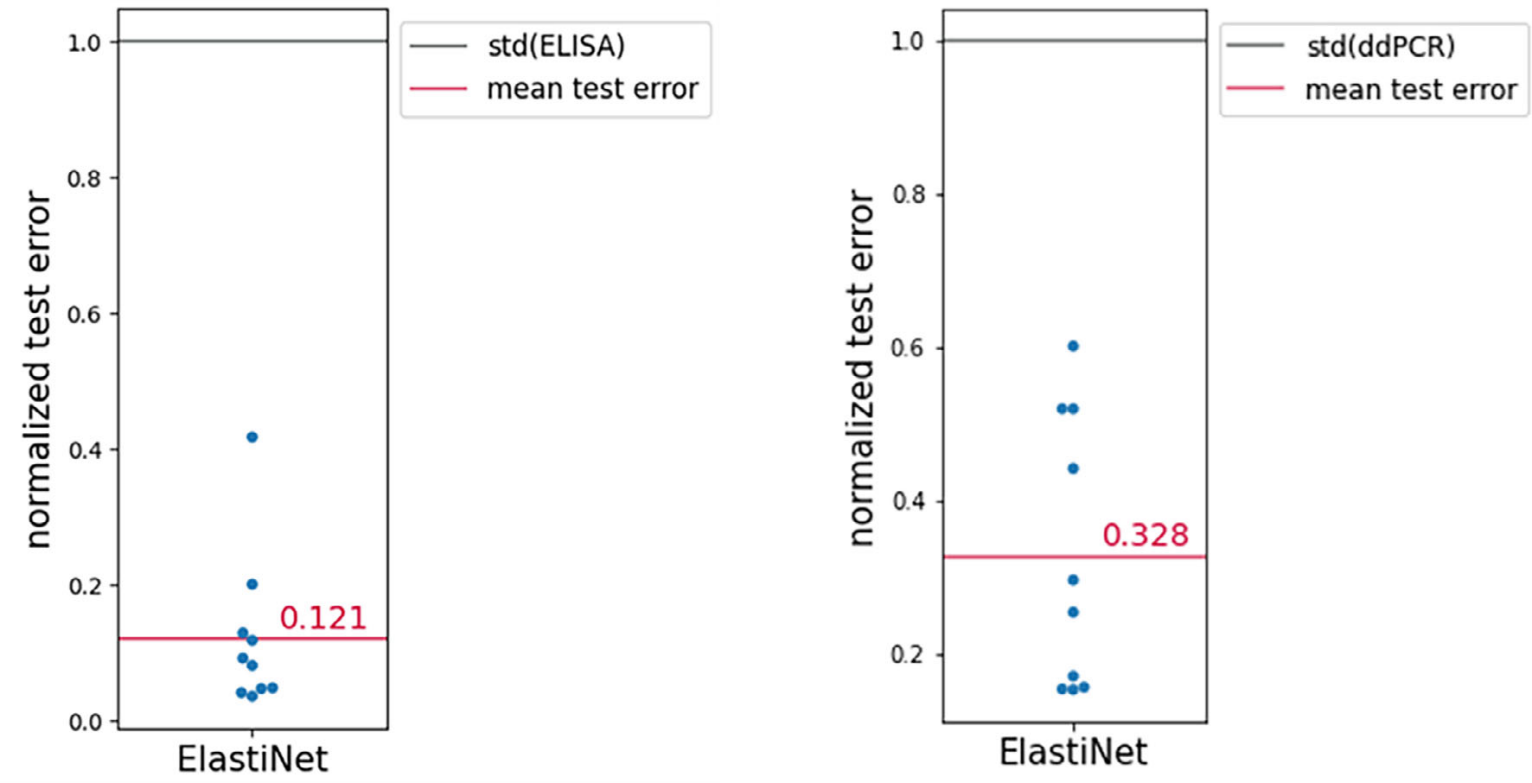
Elastic net model results for ELISA and ddPCR respectively for shaker flask campaign C1. LOOCV was used to evaluate model performance. Replicate pairs are treated as one group for the purpose of the cross‐validation. The rRMSE is depicted for every pair of replicates in the validation set.

**FIGURE 4:**
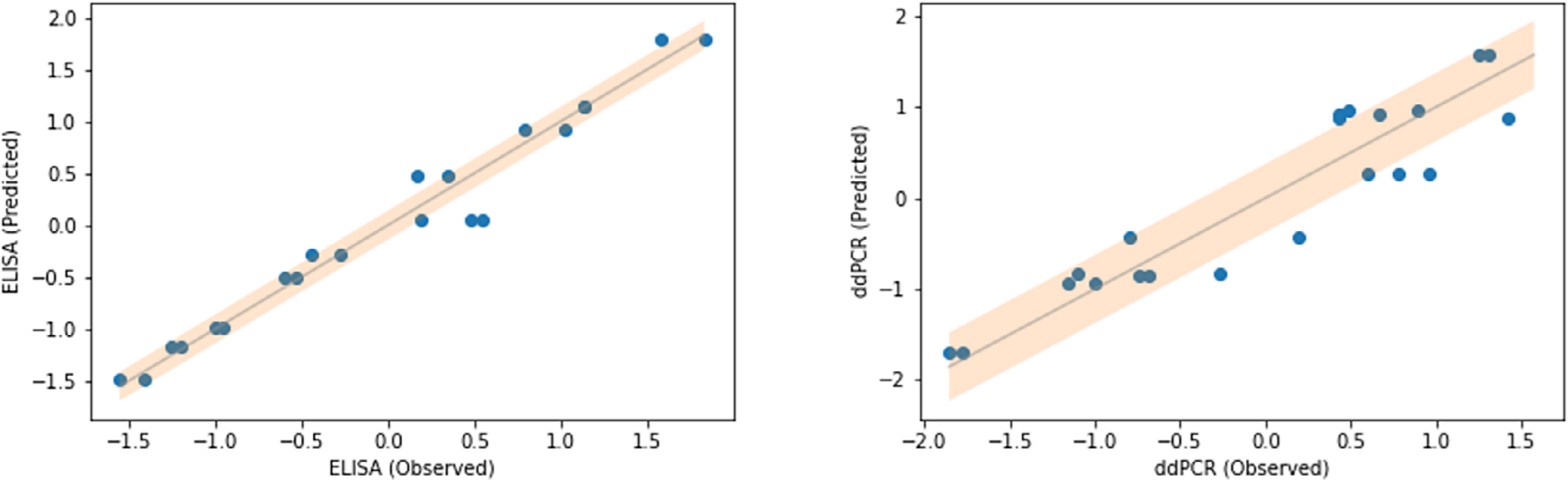
Observed versus Predicted plots for the Elastic Nets. The experiments in the validation set are listed individually. The Depicted values for ELISA and ddPCR are Z‐score normalized.

For the Elastic Net, up to 2nd degree polynomials were included. Using cross‐validation, for ELISA a rRMSE of 0.12 was obtained on the validation set. For ddPCR, a rRMSE of 0.33 was obtained on the validation set. It can be seen in Figures 3 and 4 that the model performance is satisfactory, as would have been expected provided the nature of the underlying design, and that hence this type of model can be exploited for process optimization.

#### 3.1.2 Optimization of Ambr campaign C1

**FIGURE 5:**
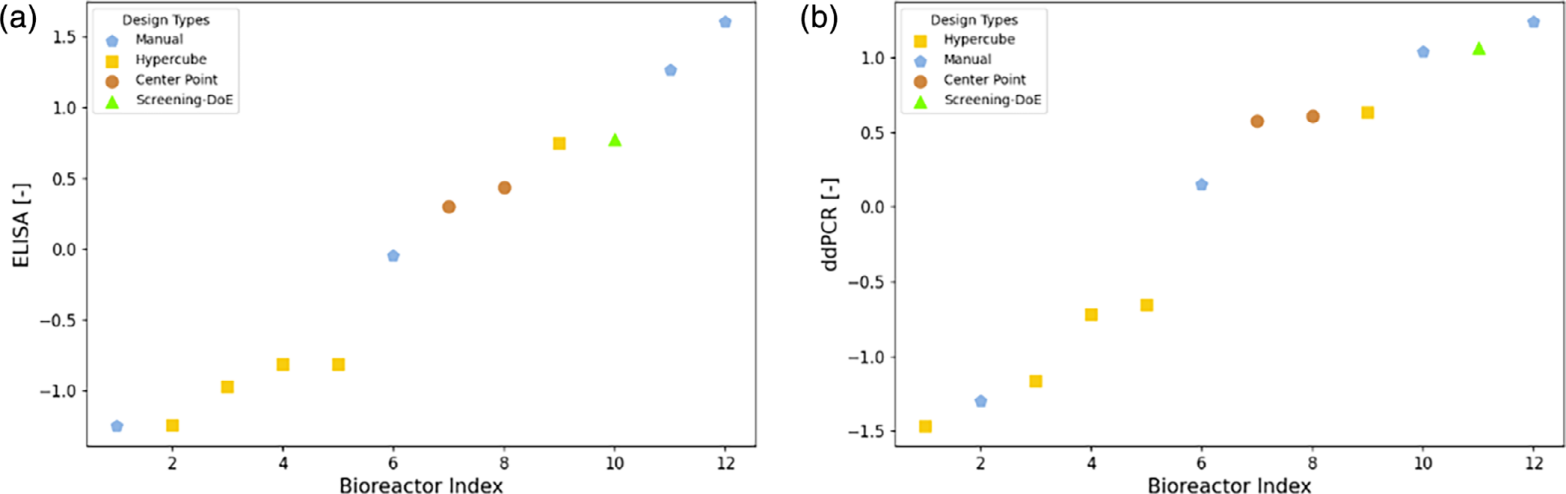
Experimental results of ELISA (left) and ddPCR (right) for campaign C1. The analytics data was normalized according to Z‐Score normalization. The runs are sorted in ascending order. Each point is labeled by a marker denoting the method used for the design of the experimental condition. *Manual*: Using decision trees and a linear model; *Hypercube*: Corner point repetition from shaker flask scale; *Center Point*: Center point of hypercube; *Screening DoE – Pick*: Manual selection based on the screening DoE.

The best Elastic Net model was used to select the conditions that give rise to the highest ddPCR value, in addition to the two suggestions obtained from the Bayesian optimization approach. The obtained experimental results of ELISA and ddPCR are depicted in Figure 5. The results for ELISA and ddPCR are overall very similar in terms of the ranking of the methods. The optimization target was maximizing ddPCR. However, as the ELISA measurements are more precise (see e.g., the noise to signal ratio analysis in the appendix A), we consider them relevant results for the relative comparison between experimental performance. The conditions suggested by the Decision Tree and Elastic Net approaches resulted in the two best performing runs, one average and one low performing run, as shown by the *Manual* points in blue with the pentagon marker. The center points (brown circles) show average performance, mostly outperforming the *Hypercube* runs (yellow squares). This suggests that the optimal conditions are not found at the design boundaries. The condition picked from the screening DoE performs better than the average performance but is outperformed by the designs originating from the other approaches.

### 3.2 Campaign D1: From decision trees to the design of Ambr campaign D1

#### 3.2.1 Decision trees

**FIGURE 6:**
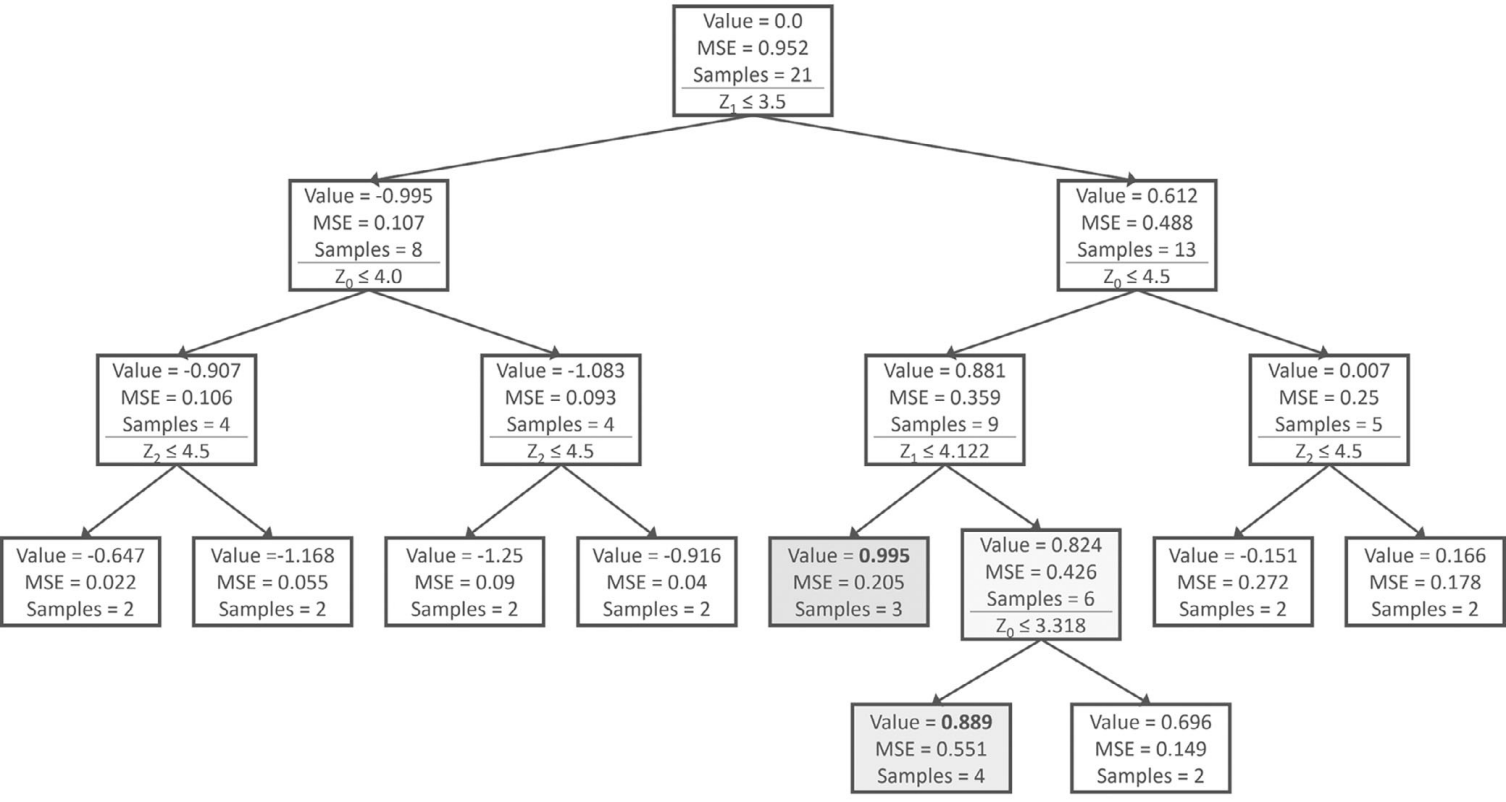
Decision tree model on shaker flask runs of project D.

Decision trees were built on the project D shaker flask data set to identify the most relevant Z‐variables and the ranges, the obtained tree is shown in Figure 6. For the highest values of ddPCR, Z1 is higher than 3.5 (just as it was the case for project C) and Z0 is lower than 4.5. In general, Z2 above 4.5 seems to be resulting in greater titer results, although no definite conclusions may be drawn. For the second highest set of runs, Z0 is lower than 3.318. These results are very similar to those obtained for the shaker flask data set in project C, which hints at high similarity of the process behavior in the two projects, perhaps allowing to transfer conclusions from one project to the next.

Developing models based on data for campaign D1 that achieved a sufficiently low rRMSE to deem the model usable for optimization was not possible due to the high noise to signal ratios, see appendix A.

#### 3.2.2 Designing of Ambr campaign D1

Insights from Decision Trees, the shaker flask runs, and initial Ambr run of project C were utilized to design the first set of Ambr runs for project D.

Due to the high replicate variability (see appendix A, section 6.2), five replicates were planned to ensure the possibility of a replicate analysis in the Ambr data set, among those replicate conditions at the center point.

To maximize cross‐project learning from C to D, the same five corner point runs, that were run in the shaker flasks of both project C and D, as well as in the Ambr campaign C1, were selected to be re‐run in the first Ambr campaign of project D, one of them as a replicate. The center point was also again run as a replicate. Additional runs for the design were manually selected to explore previously uncharted regions of the design space that has not been covered by the Ambr C1 campaign. The Z‐variable *Z3*, which did not yield valuable insights in earlier runs due to large noise to signal ratios, was omitted to streamline the investigation, and focus on the most relevant factors.

The best and second‐best performing conditions from the Ambr campaign C1 were added as replicate runs. This was based on the observation of the very similar ddPCR values of project C and D in the shaker flask data, as well as the Decision Trees delivering the conclusion that similar ranges of the transfection mixture components are beneficial for either project. This was also anticipated to further increase the learnings across data sets, enabling comparison of projects C and D in the Ambr scale.

**FIGURE 7:**
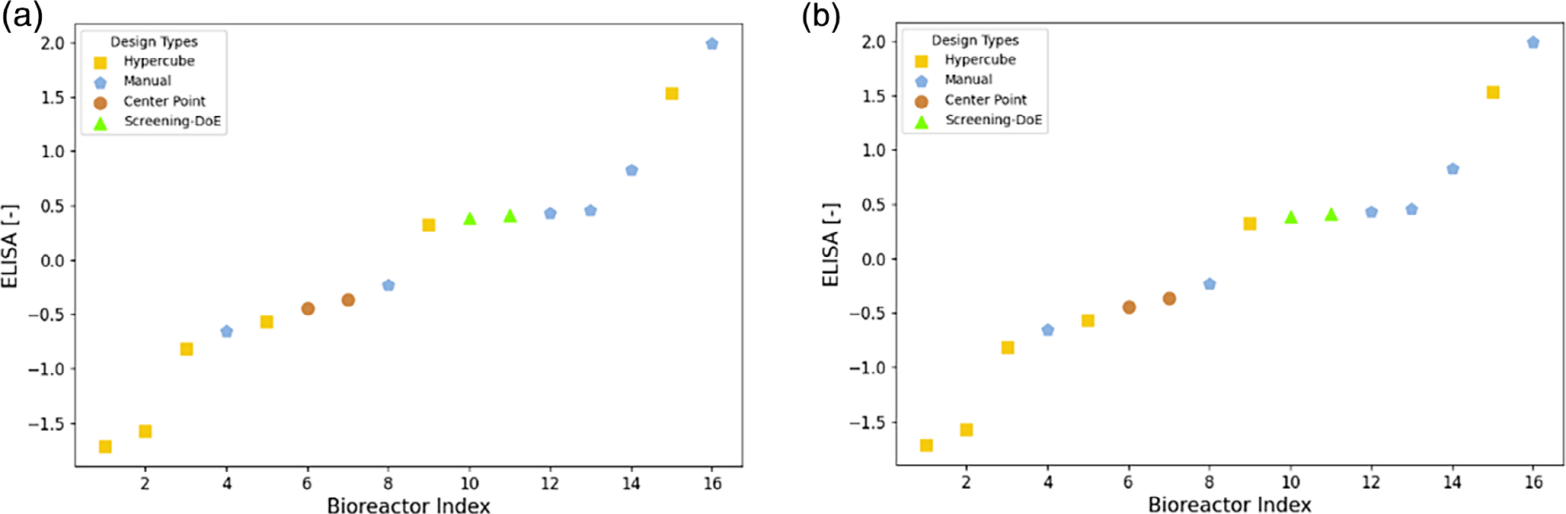
Experimental results of ELISA (left) and ddPCR (right) for campaign D1. The analytics data was normalized according to Z‐Score normalization. The runs are sorted in ascending order. Each point is labeled by a marker denoting the rationale behind the selection of the experimental conditions. *Manual*: Using decision trees; *Hypercube*: Corner point repetition from shaker flask scale; *Center Point*: Center point of hypercube; *Screening DoE – Pick*: Manual selection based on the screening DoE for internal control of process performance.

The experimental results obtained for campaign D1 are displayed in Figure 7. The center point run was performed as a replicate as a reference and measure for process variability as well as campaign‐to‐campaign variability. In this case, already many of the experimental conditions that were found using cross‐project modeling (blue pentagon points) are outperforming the center point. Therefore, as the Decision Tree predicted, conditions that perform well in project C also result in high titer in project D. The Screening‐DoE pick (green upwards triangles) also produced greater titer than the center point, but not as much as the *Manual* points that were picked using the Decision Trees. Since this was the first iteration in the Ambr scale of project D, some corner points (*Hypercube*) were repeated from the shaker flask scale. In general, these perform worse than the center point, as was the case in the Ambr C1 campaign. Although some reordering is present due to increased measurement noise for ddPCR, the results of both ddPCR and ELISA deliver the same message, namely that by leveraging the project C data for modeling, experimental conditions are found that outperform the benchmark approach.

### 3.3 Campaign C2: From a full model to optimal designs for Ambr campaign C2

#### 3.3.1 Full modeling for campaign C2

**FIGURE 8:**
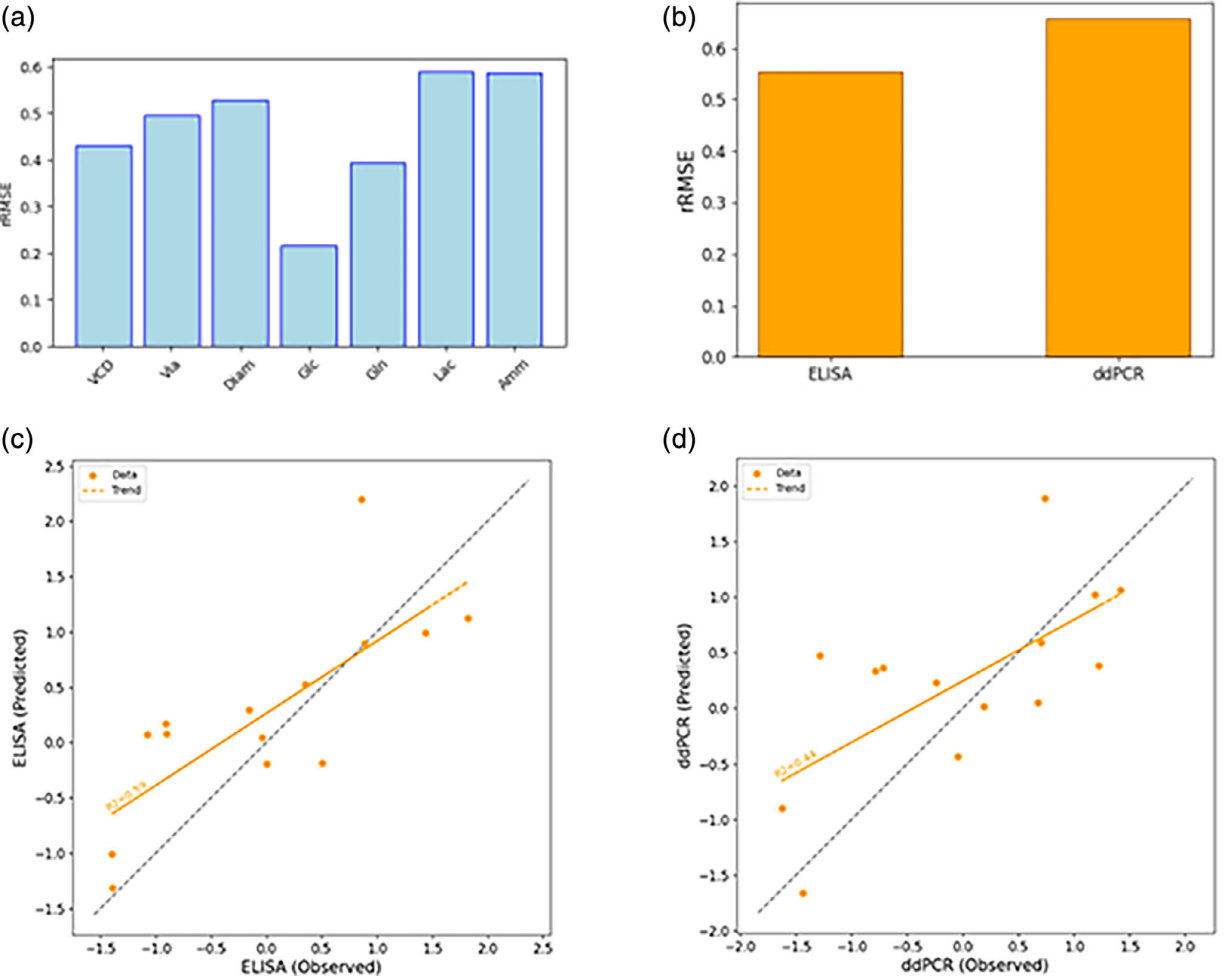
Results of modeling for the C2 campaign design. Figure (a) shows the rRMSE of the propagation model for all X variables in the model, (b) the rRMSE of the full model for ELISA and ddPCR, and figures (c) and (d) depict the observed versus predicted values of the full model for ELISA and ddPCR, respectively. The latter demonstrates the model's capability to predict the viral titers from initial conditions and design parameters with sufficient accuracy.

For the design of campaign C2, the dataset from the Ambr C1 campaign was available. In contrast to the shaker flask runs, time series data for the state variables were available for the data of the Ambr runs, enabling the training of both propagation and historical models. Only the Ambr data was used for the training and testing. The modeling outcomes are depicted in Figure 8. For most of the state space variables, the obtained rRMSE values are about 0.5 and the model performance deemed satisfactory for optimization. Also, ELISA and ddPCR predictions of the full model exhibit an adequate degree of precision, especially when considering the significant noise to signal ratios, see appendices. The plots comparing observed versus predicted values reveal that the full model successfully captures the underlying trends for ELISA and ddPCR. Notably, the prediction of ELISA shows superior performance. This is consistent with expectations, given the relatively lower measurement error associated with ELISA.

#### 3.3.2 Optimization of conditions for campaign C2

After having tested the generalization capability of the model, the same model structure was retrained using all data of the C1 campaign in the training set to increase the coverage of the process design space. The resulting models were used for optimization. The objective function was to maximize ddPCR and several conditions were derived by adapting the objective function and risk level as described in the method section.

**FIGURE 9:**
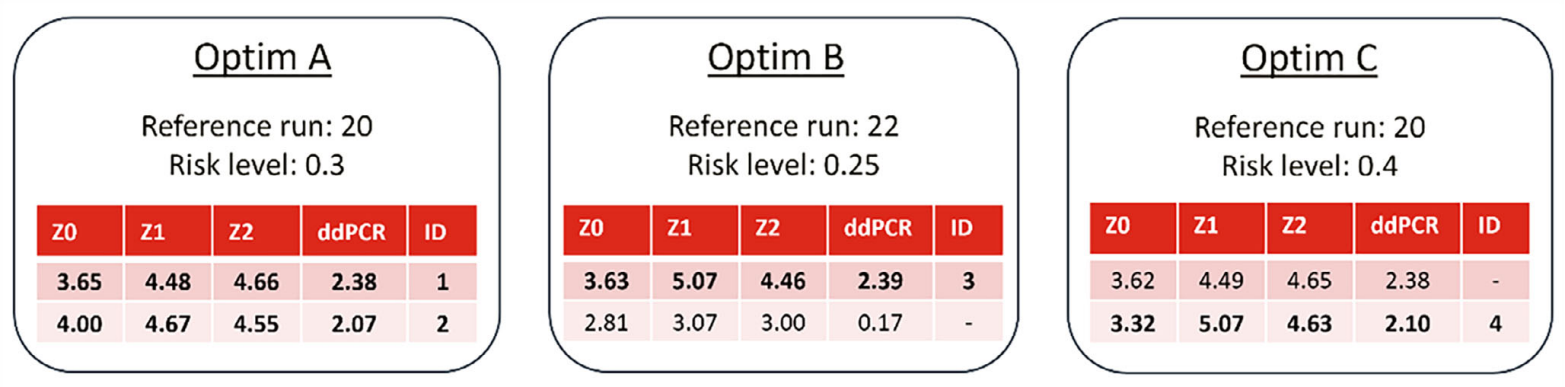
Optimizer suggestions of the experimental conditions of the main process design parameters Z0, Z1 and Z2 for campaign C2. The model predicted ddPCR for these conditions given the reference run is displayed in the fourth column. Risk level and reference run were varied. Rows with bold font were selected conditions and labeled with numeric identifiers.

In Figure 9, the process design parameters suggested by the optimizer are shown. Three distinct optimizations were performed (denoted by A, B and C), where the reference run and risk level were varied. The first row in each optimization corresponds to the design and titer values of the highest predicted titer run (most exploitative), whereas the design and titer values of the second row balance exploitation more with exploration compared to the highest titer result (greater risk level). The suggested values for *Z0*, *Z1* and *Z2* required rounding, due to accuracy constraints in the preparation of the transfection mixture. From these proposed conditions, four were performed as indicated by the bold font. The most exploitative suggestion for optimization A and C is almost identical, therefore only one of these conditions was added to the design of campaign C2. The second proposed run of optimization B was not selected, as the predicted titer was much lower compared to the rest.

**FIGURE 10:**
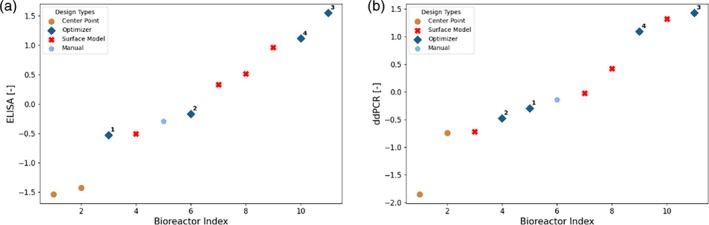
Z‐Score normalized experimental analytics results (left: ELISA, right: DdPCR) for campaign C2. The analytics results are sorted in ascending order. Each point is labeled by a marker denoting the rationale behind the selection of the experimental conditions. *Manual*: Adaptation based on optimizer suggestion; *Optimizer*: Design parameters directly proposed by the Bayesian optimizer; *Center Point*: Center point of hypercube; *Surface Model*: State of the art methodology.

The experimentally obtained results for ELISA and ddPCR are shown in Figure 10. The replicates of the center point are consistent for ELISA, and the obtained values are rather low. The results obtained with full model are significantly greater, showing that the model‐based design allows to consistently find new experimental conditions that yield higher titer. For both ELISA and ddPCR, the optimizer found the experimentally best performing condition. A priori, it was not expected that the performance of all full model derived conditions would deliver highest values, as with the chosen objective function and risk levels the process parameter design space was explored. The experimental results obtained for the design derived with the Response Surface Modeling also show high titer values. However, the best conditions were derived from the full model using the described optimization approach, that is, the greatest value obtained with the optimizer is 7.0% or 4.4% greater than the ones obtained with response surface modeling for ELISA and ddPCR, respectively.

### 3.4 Campaign D2: From transfer learning models to optimal process conditions

#### 3.4.1 Transfer learning models for D2

In the design of campaign D2, there was the opportunity to leverage data from all preceding campaigns. The focus was on modeling the process dynamics using the full model, wherefore only data from the Ambr were used. The full model was trained utilizing data from the Ambr campaigns C1, C2 and D1. The rationale behind this approach was the potential for cross‐project learning in case the projects showed sufficient similarities and hence, that a model trained on data from both projects would have an enhanced predictive accuracy through knowledge transfer.

To assess the merits of using the historical data of C1, C2 and D1 campaigns, we performed a comparison of model performance for two scenarios assuming different training data sets. In the first scenario, models were trained solely on D1 experiments, predicting a withheld D1 run. In the second scenario, the models were trained using D1 runs along with the entire dataset from campaigns C1 and C2. Evaluation followed a leave‐one‐out rotation on all D1 runs. Normalization of the variables was applied within campaigns for both X and Y variables accounting for differences between campaigns.

**FIGURE 11:**
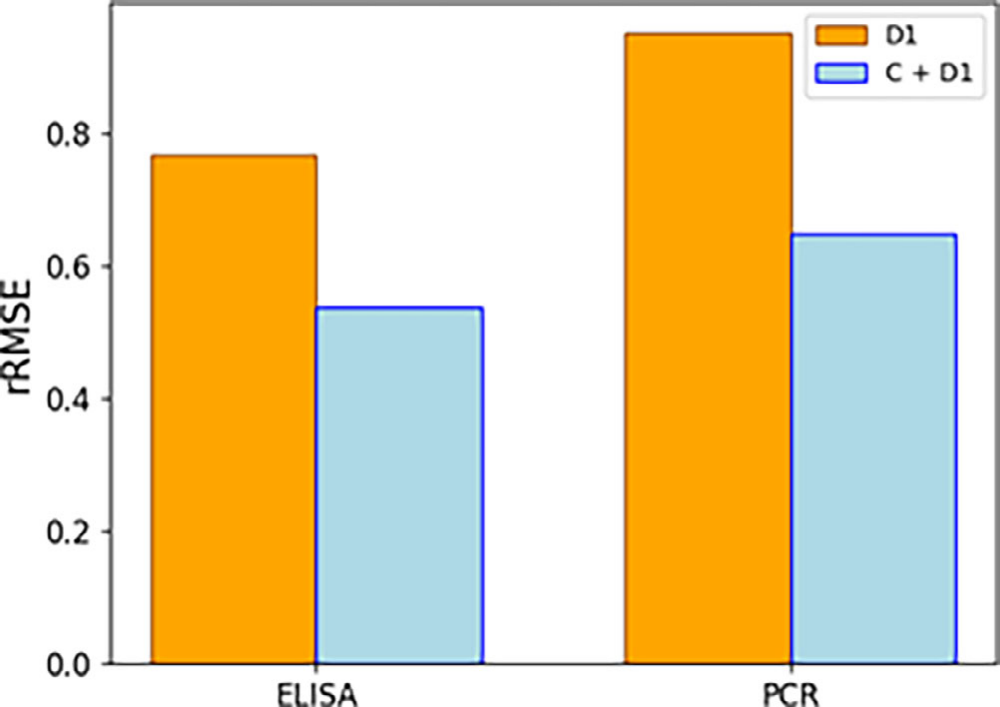
rRMSE results of modeling the D1 campaign without (orange) and with (blue) project C data, respectively. The addition of project C data provides clear benefit in terms of predictive performance of both viral titers, as seen by the decrease in rRMSE.

The results in terms of prediction errors obtained with the full model with and without the project C, data are illustrated in Figure 11. The full model exhibits a reduced rRMSE for ELISA and ddPCR when including data from campaigns C1 and C2, demonstrating successful knowledge transfer between the projects. It is worth highlighting that project D demonstrated pronounced noise to signal ratios at the shaker flask scale, which hampered the construction of meaningful models from these data alone. The magnitude of these noise to signal ratios might also reduce the performance of models relying solely on D1 Ambr data. The cross‐campaign knowledge transfer seems to alleviate these shortcomings, facilitating improved predictions.

#### 3.4.2 Optimization of campaign D2 Ambr runs

As for campaign C2, the same model structure was retrained with all runs of campaigns C1, C2 and D1 in the training set. The objective function was to maximize ddPCR titer.

**FIGURE 12:**
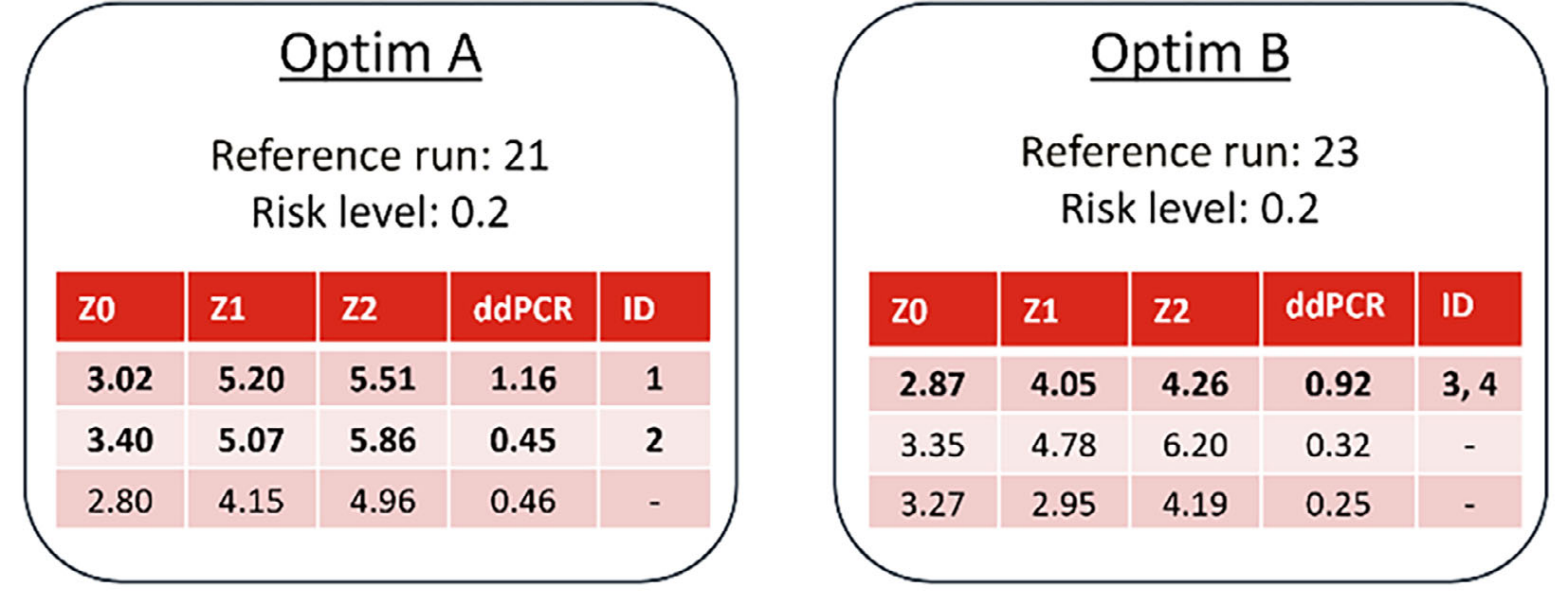
Optimizer suggestions of the experimental conditions of the main process design parameters *Z0*, *Z1* and *Z2* for campaign D2. The model predicted ddPCR for these conditions given the reference run is displayed in the fourth column. Risk level and reference run were varied. Rows with bold font were selected conditions and labeled with numeric identifiers.

Figure 12 shows the proposed experimental conditions by the optimizer, for two separate optimizations that were performed with different settings. The risk level was held at 0.2 and the reference run was varied. For each optimization the most exploitative suggestion has a much higher predicted titer than when a more explorative (and risk restricted) condition is considered. Interestingly, both optimizations proposed completely different conditions. For this design of campaign D2, the highest titer predicted runs were chosen (bold font), the one from optimization B was run as a replicate. From optimization A, the second proposed run was also included in the design, as those conditions were similar to a corner point that has performed well in campaign D1 (second highest in ELISA and ddPCR, compare Figure 7).

**FIGURE 13:**
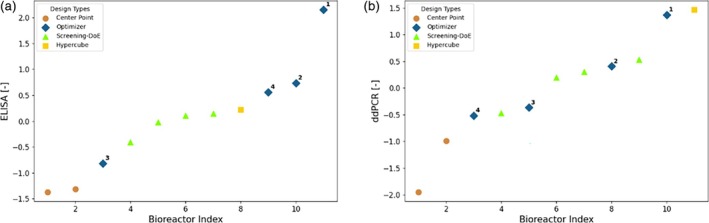
Z‐Score normalized experimental analytics results (left: (a), right: B) for campaign D2. The analytics results are sorted in ascending order. Each point is labeled by a marker denoting the rationale behind the selection of the experimental conditions. *Manual*: Adaptation based on optimizer suggestion; *Optimizer*: Design parameters directly proposed by the Bayesian optimizer; *Center Point*: Center point of hypercube; *Screening DoE – Pick*: Manual selection based on the screening DoE for internal control of process performance.

Figure 13 presents the experimental results obtained for the design of campaign D2. As in the case of campaign C2, the center point has moved to the left side of low performing points in a relative sense, highlighting the overall iterative improvement of the approaches, enabled by the increased amount of data availability. For ELISA the optimizer found two experimental conditions that give rise to the highest titer by a great margin, showing that an increased process understanding by using the full model translates into a better optimization performance, as was expected and as in line with observations in Ref. [36] The results from these conditions show clearly higher titer than the results from the internal control condition (Screening‐DoE pick) for assessing across process performance, that is, an increase in yield by 32.4%. The overall trend is the same for ddPCR, however there the best performing point is a corner point that was repeated. Due to the measurement uncertainty in ddPCR, it is difficult to make a quantitative assessment of the ddPCR results for the few available data points. For instance, the more accurate measurement of ELISA shows that the center point provided very similar titer results, whereas the same runs show a large deviation for ddPCR. Therefore, from a pure ranking perspective, the one obtained by ELISA seems more significant and was considered in the development. Considering the signal to noise ratios, the normalized relative improvements were determined as outlined in section 6.5, to assess significance of results. For ELISA, the best point found with the full model approach has a normalized relative improvement (α) of 4.17 compared to the best point of the Screening‐DoE, the mean of the points of the full model approach has α = 1.46. For ddPCR, the same best point has α = 2.07 and the mean α = 0.20, which corresponds to an increase of 10.9% in yield. Any value of α >1 exceeds the signal to noise ratio and shows significant improvement compared to the base approach. ELISA shows therefore larger and more certain improvements than ddPCR. It must be considered that in general the mean improvement is much lower than the improvement of the best point. This is because the optimization algorithm of the full model approach does not fully exploit, but also explores new regions in the process space where model uncertainty is still high.

## 4 CONCLUSION

Different mathematical models were used to maximize the production of capsids (ELISA) and in particular, genome titer (ddPCR) by manipulation of rAAV transfection conditions in two projects. The models were based on different amounts of data and knowledge available during the different stages of development. Both projects started from data obtained from shaker flask runs. These data were used to build simple models, used to inform the first campaign in Ambr runs. Subsequently, hybrid models that were describing the evolution of the process state (VCD, glucose, glutamine, glutamate, ammonia and lactate) were combined with models that predicted the ELISA and ddPCR values at the end of the cultivation, referred to as full model. Using these models, the transfection conditions were optimized, also considering the potential risk of misprediction by using a risk constraint. The results were benchmarked with the standard approach, that is, a combination of Design of Experiment and Response Surface Modeling.

It was observed that the conditions stemming from the full model across projects gave rise to higher titer values than those of the standard approach. In the case of ELISA, the relative improvement was up to 4.17 the magnitude of the signal to noise ratio. The increased insight in the process behavior obtained with the full model, was therefore exploited during the optimization, giving rise to better performance. Further, using the data from shaker flasks runs in combination with simple models seemed to allow designing more informative experiment conditions, as those could be explored effectively by the full models to find better performing process conditions. In addition, training the full model on data from the two projects showed an increase in prediction performance for ELISA and ddPCR, subsequently translating into increased process performance after optimization. Hence, in the future, one could imagine that an ever‐increasing amount of data in combination with transfer learning will further decrease the experimental effort required, which is subject to future investigations.

## AUTHOR CONTRIBUTIONS

**Claudio Müller:** Software; data curation; formal analysis; investigation; validation; writing – original draft; visualization. **Angela Botros:** Methodology; software; data curation; formal analysis; investigation; validation; writing – original draft; visualization. **Gerald Siegwart:** Investigation; writing – original draft; data curation; formal analysis; writing – review and editing. **Alexandra Umprecht:** Funding acquisition; project administration; supervision; writing – review and editing. **Susanne Heider:** Funding acquisition; writing – review and editing; supervision; investigation. **Moritz von Stosch:** Conceptualization; methodology; writing – original draft; visualization; formal analysis; project administration. **Michael Sokolov:** Methodology; validation; formal analysis; supervision; project administration; conceptualization; writing – review and editing. **Mariano Nicolas Cruz Bournazou:** Conceptualization; methodology; formal analysis; writing – original draft; writing – review and editing.

## FUNDING INFORMATION

Financial support was provided by Takeda and DataHow AG.

## CONFLICT OF INTEREST STATEMENT

The authors declare the following financial interests/personal relationships which may be considered as potential competing interests: Financial support was provided by Takeda and DataHow AG. All authors were employees of Takeda or Datahow AG at the time this study was performed.

## Figures and Tables

**FIGURE A1:**
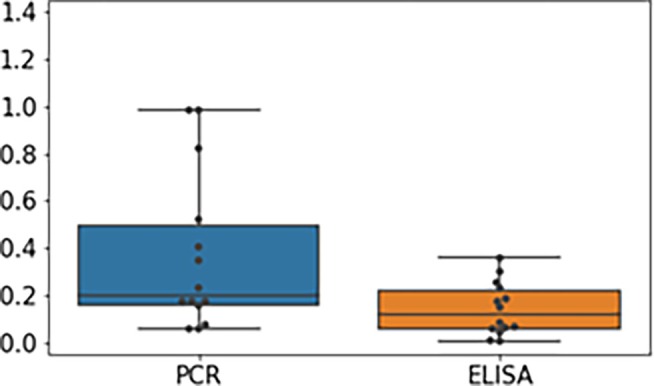
Noise to signal ratios of shaker flask experiments of project C for both ELISA and ddPCR. The noise to signal ratio for ELISA is lower than for ddPCR and has less variability, however both methods show good replicability.

**FIGURE A2:**
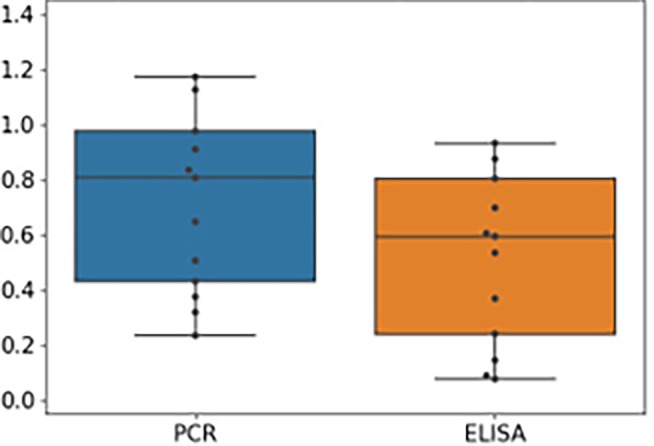
Noise to signal ratios of shaker flask experiments of project D for both ELISA and ddPCR. As for project C, the noise to signal ratio for ELISA is lower than for ddPCR. Compared to project C, the noise to signal ratios for project D are much larger, suggesting more intrinsic variability in the process.

**FIGURE A3:**
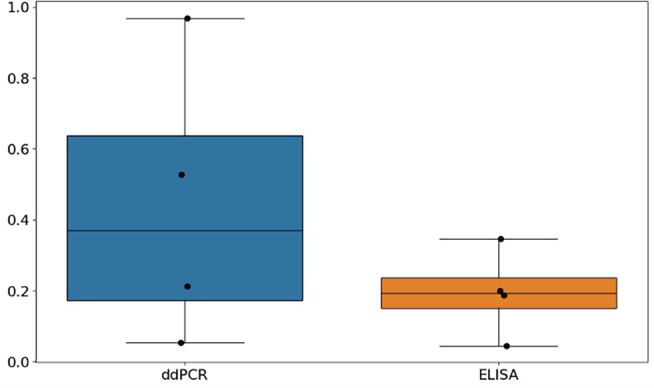
Noise to signal ratios of Ambr experiments of project C for both ELISA and ddPCR. As for project C, the noise to signal ratio for ELISA is lower than for ddPCR. Compared to project C, the noise to signal ratios for the Ambr scale for project C are comparable to shake flask.

**FIGURE A4:**
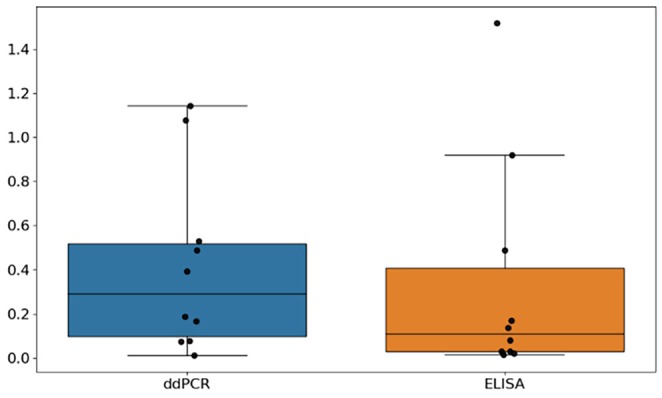
Noise to signal ratios of Ambr experiments of project D for both ELISA and ddPCR. As for project C, the noise to signal ratio for ELISA is lower than for ddPCR. Compared to project C, the noise to signal ratios for the Ambr scale for project C are comparable to shake flask.

## Data Availability

Data sharing is not applicable to this article as no new data were created or analyzed in this study.

## APPENDIX A

### A.1 NOISE‐TO‐SIGNAL RATIO SHAKER FLASK PROJECT C

In project C, an initial Design of Experiment (DoE) encompassing 25 shaker flask experiments was performed. Among these runs, factorial combinations of three primary design parameters were executed in duplicates, and the center point was run in triplicates.

An analysis of the noise to signal ratio was conducted for all experiments, where each experiment was performed at least in duplicates. For the dataset *X*, and for all pairs of replicates within the dataset, denoted as (*x*_*i*,1_,*x*_*i*,2_) ∈ *X*, which are normalized by the standard deviation of the experiments, the noise to signal ratio *e*_*i*_ was defined as:

$$e_{i}=|\mathrm{xi},1-\mathrm{xi},2|/\mathrm{std}\left(X\right).$$

For experiments performed in triplicates, three noise to signal ratios were calculated considering all pairwise combinations.

Figure A1 shows the noise to signal ratios for ELISA and ddPCR, respectively. The noise to signal ratios for the both are normalized by the standard deviation of the experiments. The results indicate that the noise to signal ratio for ELISA is relatively low, for ddPCR it is larger and has more variation.

The noise to signal ratio serves as an upper benchmark for the models, as it cannot be expected from them to predict with greater accuracy, given the variation in outputs when faced with the same inputs.

### A.2 NOISE TO SIGNAL RATIOS SHAKER FLASK PROJECT D

The shaker flask data of project D comprised of 25 experiments. All experiments were executed 2‐fold, except the center point which was run 3‐fold. In Figure A2 are the noise to signal ratios of the shaker flask data set of project D visualized. Overall, the noise to signal ratios are much higher than for project C. With this level of noise to signal ratios, modeling tasks are difficult to do in a meaningful way.

### A.3 NOISE TO SIGNAL RATIOS AMBR250 PROJECT C

Noise to signal ratios for Ambr for project C are shown in Figure A3. Four replicate groups were available. As for the shaker flask experiments, the mean variability for ELISA is much lower than for ddPCR. Compared to shaker flask of project C, the variability in the Ambr experiments is higher.

### A.4 NOISE TO SIGNAL RATIOS AMBR250 PROJECT D

For the Ambr scale of project D, 10 replicate groups were performed in duplicate, allowing for much more accurate determination of the variability compared to Ambr project C (see Figure A4). The mean signal to noise ratio of ELISA is much lower than for ddPCR. It is also significantly lower for the Ambr scale than the shaker flask scale in the case of project D. This means that for the results of campaigns D1 and D2, any improvement higher than this value is considered significant.

### A.5 NORMALIZED RELATIVE IMPROVEMENT

As signal to noise ratios are high for this process, it is not immediately apparent if a result is an objective improvement or just due to intrinsic variability or measurement uncertainty. For a given campaign the normalized the relative improvement of the full model approach compared to standard approaches is therefore of interest. It provides insight into the significance of the results and answers the question how much higher ddPCR or ELISA were achieved with the full model approach, relative to the signal to noise ratio, which is determined as outlined in section 6.1.

Normalized relative improvements were calculated according to the equation,

$$\alpha =\frac{\left(x_{f}-x_{r}\right)/\mathrm{Std}\left(X\right)}{\bar{e_{X}}}$$

Where α is the normalized relative improvement, $x_{f}$ the result of the full model approach, $x_{r}$ the result of the reference approach, $\mathrm{Std}\left(X\right)$ the standard deviation of the target values of a given project (ddPCR or ELISA) and $\bar{e_{X}}$ the median signal to noise ratio of target *X* (ddPCR or ELISA) for the respective project, as given by the boxplots in sections 6.3 and 6.4. Two α values were considered, the first with respect to only the best point of either approach, the second considering the mean of all designed experiments with this approach for a given campaign.
